# 2-Bromopalmitate Reduces Protein Deacylation by Inhibition of Acyl-Protein Thioesterase Enzymatic Activities

**DOI:** 10.1371/journal.pone.0075232

**Published:** 2013-10-02

**Authors:** Maria P. Pedro, Aldo A. Vilcaes, Vanesa M. Tomatis, Rafael G. Oliveira, Guillermo A. Gomez, Jose L. Daniotti

**Affiliations:** 1 Centro de Investigaciones en Química Biológica de Córdoba, Departamento de Química Biológica, Facultad de Ciencias Químicas, Universidad Nacional de Córdoba, Córdoba, Argentina; 2 Queensland Brain Institute, The University of Queensland, Queensland, Australia; 3 Division of Molecular Cell Biology, Institute for Molecular Bioscience, The University of Queensland, St. Lucia, Brisbane, Queensland, Australia; University of South Florida College of Medicine, United States of America

## Abstract

S-acylation, the covalent attachment of palmitate and other fatty acids on cysteine residues, is a reversible post-translational modification that exerts diverse effects on protein functions. S-acylation is catalyzed by protein acyltransferases (PAT), while deacylation requires acyl-protein thioesterases (APT), with numerous inhibitors for these enzymes having already been developed and characterized. Among these inhibitors, the palmitate analog 2-brompalmitate (2-BP) is the most commonly used to inhibit palmitoylation in cells. Nevertheless, previous results from our laboratory have suggested that 2-BP could affect protein deacylation. Here, we further investigated in vivo and in vitro the effect of 2-BP on the acylation/deacylation protein machinery, with it being observed that 2-BP, in addition to inhibiting PAT activity in vivo, also perturbed the acylation cycle of GAP-43 at the level of depalmitoylation and consequently affected its kinetics of membrane association. Furthermore, 2-BP was able to inhibit in vitro the enzymatic activities of human APT1 and APT2, the only two thioesterases shown to mediate protein deacylation, through an uncompetitive mechanism of action. In fact, APT1 and APT2 hydrolyzed both the monomeric form as well as the micellar state of the substrate palmitoyl-CoA. On the basis of the obtained results, as APTs can mediate deacylation on membrane bound and unbound substrates, this suggests that the access of APTs to the membrane interface is not a necessary requisite for deacylation. Moreover, as the enzymatic activity of APTs was inhibited by 2-BP treatment, then the kinetics analysis of protein acylation using 2-BP should be carefully interpreted, as this drug also inhibits protein deacylation.

## Introduction

Fatty-acylated peripheral proteins, such as members of the small G-protein Ras family, the neuronal proteins PSD-95 and growth-associated protein-43 (GAP-43) [1]–[5], are synthesized in the cytosol and post-tranlationally modified by different lipid moieties [6]–[8], with these modifications governing their membrane association and membrane subdomain segregation, as well as their trafficking, function and stability [9], [10].

Despite the many post-translational lipid modifications of proteins that have been achieved, including isoprenylation and myristoylation, the addition of fatty acid to the sulfhydryl group of a cysteine to form a thioester bond (S-acylation, often referred as palmitoylation) is the only known readily reversible linkage that has a much shorter half-life than that of the protein itself [11]–[16]. Consequently, S-acylation can operate as a switch, regulating not only the protein-membrane binding affinity and segregation, but also modulating the proteins biological activities [17]–[19]. S-acylation is catalyzed by protein acyltransferases (PATs) whereas deacylation requires acyl-protein thioesterases (APTs).

PATs have been identified both in yeast and mammals [20], [21] and have a 51-amino-acid domain containing a DHHC (aspartate-histidine-histidine-cysteine) motif and a high abundance of cysteine residues. Additionally, a novel and conserved 16-amino-acid motif present at the cytosolic C-terminus of PATs was recently identified to be required for protein acylation mediated by PAT [22]. The mammalian and yeast genomes encode up to 24 and 7 PATs, respectively, which are integral membrane proteins predicted to contain 4 to 6 transmembrane domains. S-acylation has been reported to occur in several membrane compartments [1], [23]–[25] with apparent substrate selectivity. However, S-acylation of semisynthetic substrates is detectable only in the Golgi complex with substrate specificity not being essential for the reacylation step [26], [27].

The enzymes mediating deacylation have not been characterized as extensively as the PATs, and only two cytosolic APTs have been described to date: APT1 and APT2, which were originally isolated as lysophospholipases and later demonstrated to be effective as protein thioesterases [19], [28]–[30]. Although APTs mediate fatty acid turnover on many cytoplasmic proteins, such as heterotrimeric G protein α subunits, endothelial nitric-oxide synthase, SNAP-23, GAP-43 and H-Ras, it has been demonstrated that APT1 and APT2 are more selective. For instance, caveolin and GAP-43 are not deacylated by APT1 [29], [31], calcium-activated potassium channel is not deacylated by APT2 [32] and not all substrates are deacylated with the same efficiency [33].

After the discovery and initial characterization of PATs and APTs, it has become of increasing interest to develop pharmacologic inhibitors for these enzymes. This is based on the necessity to modulate the localization and activity of many important intracellular acylated proteins, several of which are involved in pathological processes, with most of the research in this area having been focused on the H- and N-Ras proteins, as they play a causative role in melanoma, leukemia and cancers of the liver and kidney. However, lipid based inhibitors of S-acylation have been limited to 2-brompalmitate (2-BP), cerulenin and tunicamycin [34], among which, 2-BP is the most commonly used to inhibit S-acylation in cells [5], [29], [35], [36] and PAT activity in vitro [37], [38]. More recently, several non-lipid inhibitors have also been identified by high throughput screening [39] and are now being further characterized [38].

The palmitate analog 2-BP is a electrophilic α-brominated fatty acid which has been widely used to inhibit the palmitoylation of several proteins, including the H-Ras, GAP-43 and Rho family kinases [5], [36], [40]. In fact, 2-BP acts as a general inhibitor of palmitate incorporation and does not appear to selectively inhibit the acylation of specific protein substrates. It was also found to inhibit fatty acid CoA ligase and other enzymes involved in lipid metabolism, thus affecting the levels of intracellular palmitoyl-CoA, a necessary donor substrate for S-acylation [41].

During the course of previous experiments carried out in our laboratory to investigate the mechanisms of GAP-43 membrane affinity, it was observed that PAT inhibition with 150 µM 2-BP completely eliminated acylation of newly synthesized GAP-43, and consequently its binding to the membranes. However, at steady-state conditions, 2-BP treatment did not modify the acylation state or membrane binding properties of GAP-43 [5], thereby strongly suggesting that membrane-associated GAP-43 was not being deacylated and that 2-BP not only inhibits PATs but also APT activity. Some subsequent experiments were therefore conducted at lower concentrations of 2-BP in order to inhibit the PATs and minimally affecting the deacylating enzyme activities [29].

Taking into consideration that 2-BP is widely used to inhibit protein palmitoylation and that it is sometimes referred to as a “specific” inhibitor of acylation, we considered it essential to investigate further the in vivo and in vitro effects of 2-BP on the acylation/deacylation protein machinery, by paying particular attention to the deacylation enzymatic process. Briefly, we observed that 2-BP in vivo, in addition to inhibiting PAT activity, also perturbed the acylation cycle of GAP-43 at the level of deacylation. Next, the study was extended to evaluate the ability of 2-BP to affect the enzymatic activities of recombinant human APT1 and APT2 in vitro. Interestingly, both thioesterases showed a significant profile of inhibition by 2-BP. On the basis of these results, we concluded that 2-BP treatment inhibits the APT1 and APT2 activities both in vitro and in vivo. This not only implies that the kinetics analysis of protein acylation using 2-BP should be carefully interpreted because this drug also inhibits protein deacylation, but also suggests that the 2-BP moiety can be used as a model for the rational design of new drugs that may be able to modify the oncogenic signaling of acylated proteins (i.e. N- and H-Ras), which may lead to the development of new therapies for cancer.

## Results

### 2-BP Inhibits PAT Activity and hence the Membrane Association of a Single Acylated Protein

To investigate in vivo the effect of 2-BP on the acylation/deacylation protein machinery, we set up a direct method for acylation/deacylation readout using a monoacylated mutant of GAP-43, which requires a single acylation event for its membrane association. GAP-43 is a dually palmitoylated protein found in cysteine residues at positions 3 and 4. As previously observed, the acylation motif (MLCCMRRTKQVEK) of GAP-43 (^N13^GAP-43) fused to the N-terminal domain of spectral variants of green fluorescent protein (GFP) localized at steady-state at the recycling endosome, plasma membrane and trans Golgi network (TGN) [5], [29]. The point mutation at Cys^3^ in ^N13^GAP-43 [^N13^GAP-43(C3S)] caused an accumulation at the cytosol and TGN, which disrupted recycling endosome association but did not affect plasma membrane association [5], [29].

**Figure 1:**
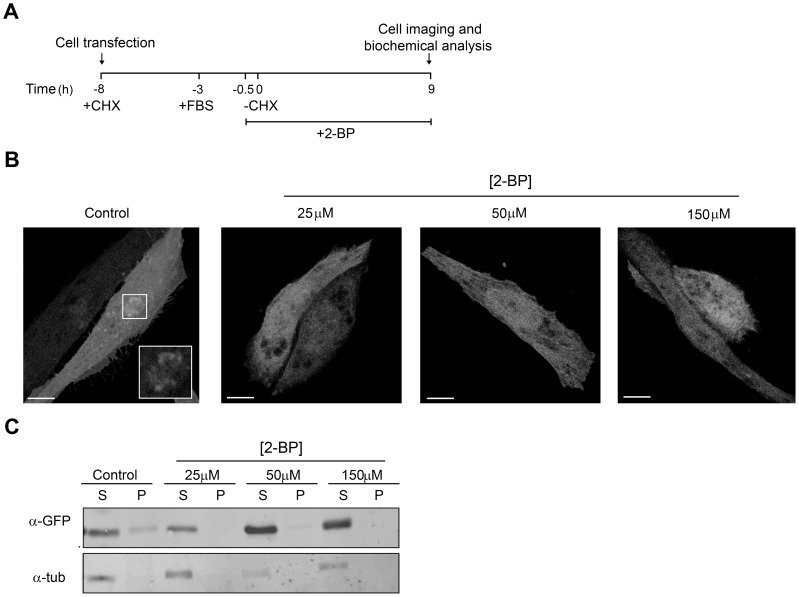
2-BP inhibits acylation and membrane association of newly synthesized ^N13^GAP-43(C3S). **A**) Schematic representation of the experimental procedure used in **B** and **C**. The CHO-K1 cells were transfected at −8 h with the plasmid encoding ^N13^GAP-43(C3S)-YFP. At 30 min before CHX withdrawal (-CHX), cells were incubated with 25, 50 or 150 µM 2-BP or DMSO (vehicle, Control). At 0 h, CHX was removed and cells were further incubated with 2-BP at the concentrations indicated above, or with DMSO, at 37°C for 9 h. Finally, cells were analyzed by confocal fluorescent microscopy or subjected to biochemical assays. **B**) Representative images showing the effect of 2-BP or DMSO (Control) on the TGN association of ^N13^GAP-43(C3S). The fluorescent signal from YFP was pseudocoloured gray. The inset shows details of the boxed area at a higher magnification. Scale bars: 5 µm. **C**) After treatment with 2-BP, CHO-K1 cells transiently expressing ^N13^GAP-43(C3S)-YFP were lysed, ultracentrifuged and the supernatant (S) and pellet (P) fractions were recovered. Proteins from these fractions were western blotted with an antibody to GFP (α-GFP) and α-tubulin (α-tub).

The effect of 2-BP on the acylation of newly synthesized ^N13^GAP-43(C3S) was tested after synchronized protein expression in Chinese hamster ovary (CHO)-K1 cells (Fig. 1A). In control conditions, the newly synthesized GAP-43(C3S)-YFP associated to TGN membranes due to S-acylation by TGN resident PATs [5]. However, treatment with 25, 50 and 150 µM 2-BP inhibited membrane association, especially to TGN, of ^N13^GAP-43(C3S) (Fig. 1B), an observation which is in agreement with the notion that 2-BP inhibits protein acylation. To support the confocal microscopy experiments, membrane association and the extent of the post-translational modification of ^N13^GAP-43(C3S) were determined at the same experimental conditions by ultracentrifugation. It was found that 2-BP, when compared to control conditions, caused a significant decrease of membrane bounded-^N13^GAP-43(C3S) at all analyzed concentrations (25, 50 and 150 µM) (Fig. 1C). Overall, these biochemical results are in close agreement with those obtained by confocal microscopy analysis and clearly indicate that 2-BP inhibits PAT activity and consequently the membrane binding of ^N13^GAP-43(C3S).

### 2-BP Perturbs the Deacylation Kinetics of Monoacylated GAP-43

**Figure 2:**
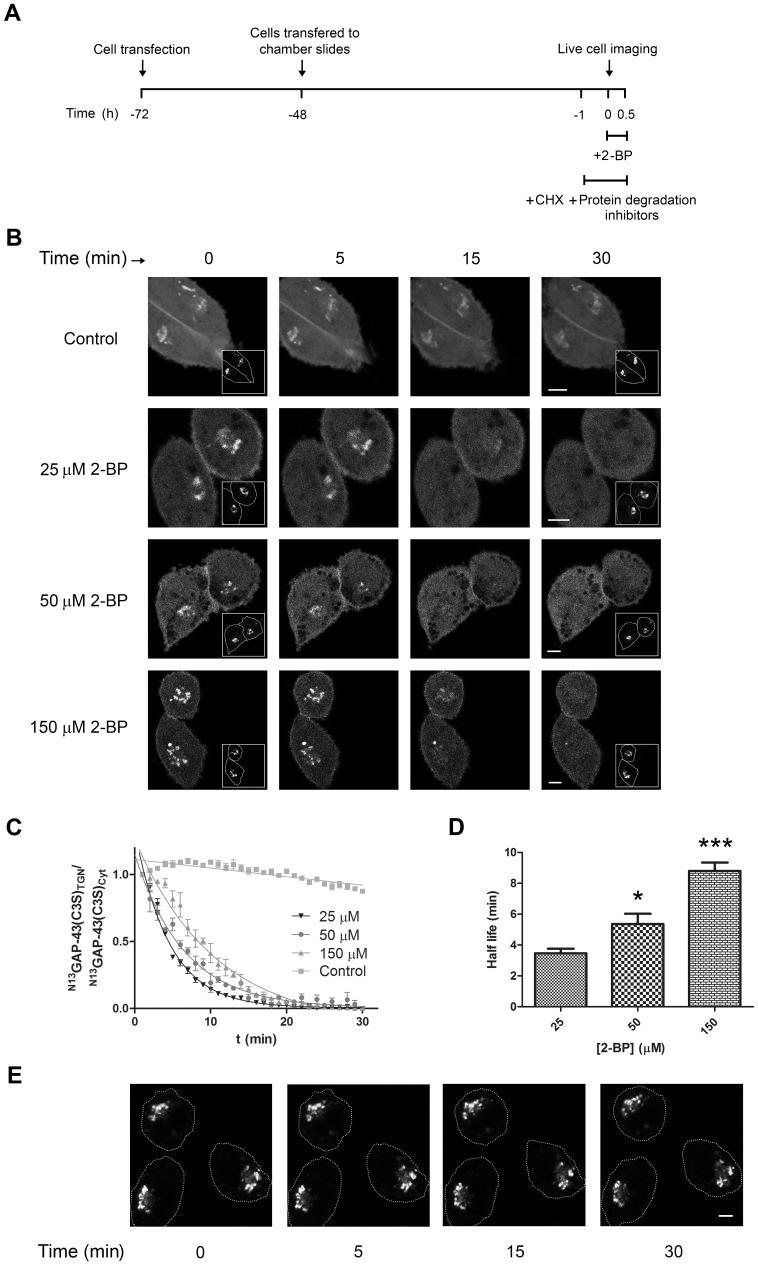
Deacylation kinetic of ^N13^GAP-43(C3S) at different doses of 2-BP. **A**) Schematic representation of the experimental procedure used in **B**, **C** and **E**. The CHO-K1 cells expressing ^N13^GAP-43(C3S)-YFP, 72 h after transfection, were treated at 20°C with 25, 50 or 150 µM 2-BP or DMSO (Control) in the presence of CHX and protein degradation inhibitors which were added 1 h before imaging and during all the experiments. The ^N13^GAP-43(C3S) subcellular distribution was analyzed by live cell confocal microscopy. **B**) Representative images showing the effect of different doses of 2-BP or DMSO (vehicle, Control) on the TGN-membrane association of ^N13^GAP-43(C3S)-YFP. The fluorescent signal from YFP (pseudocoloured gray) at 0, 5, 15 and 30 min after 2-BP or vehicle addition is shown. The insets show the expression of the TGN marker GalNAc-T-CFP (pseudocolored gray). Cell boundaries (white lines) are indicated. **C**) Quantification of the images shown in **B** (for details see Materials and methods). Curves were fitted to the exponential decay function for each data set, and data are expressed as means±SEM for a representative experiment from nine independent ones. **D**) The half-life for deacylation at each 2-BP dose calculated from the **C** data (n = 6). (*p<0.05; ***p<0.0001; compared to 25 µM). **E**) Representative images showing the effect of 50 µM 2-BP on the TGN-membrane association of GalNAc-T-YFP over time. The fluorescent signal from YFP (pseudocoloured gray) at 0, 5, 15 and 30 min after 2-BP addition is shown. Scale bars: 5 µm.

Having demonstrated that 2-BP inhibited PAT activity in vivo at a range of concentrations between 25 and 150 µM, we next investigated whether the α-brominated fatty acid could also perturb the deacylation kinetics of monoacylated GAP-43 at the same concentrations. Thus, CHO-K1 cells transiently coexpressing ^N13^GAP-43(C3S) and GalNAc-T (TGN marker) were treated with 25, 50 and 150 µM 2-BP (2-BP) or dimethylsulfoxide (DMSO, Control) and the GAP-43 subcellular distribution was monitored at different times by live cell confocal fluorescent microscopy, with cycloheximide (CHX) and protein degradation inhibitors being incorporated to the culture medium 1 h before and during 2-BP treatment (Fig. 2A). In control cells, the amount of TGN-membrane association of GAP-43(C3S) did not significantly change over time, whereas in cells treated with 25 µM 2-BP, the TGN-associated fraction of GAP-43 significantly decreased over time with a half-life of 3.5±0.1 min (Fig. 2B, C and D; and movie S1). Interestingly, a significant reduction in the ^N13^GAP-43(C3S) depalmitoylation rate was clearly observed at higher 2-BP concentrations (Fig. 2B). The calculated half-life of the TGN-associated fraction of GAP-43(C3S) in cells treated with 50 and 150 µM 2-BP was 5.4±0.3 and 8.8±0.2 min, respectively (Fig. 2C and D; and movie S1). However, the observed decrease of the TGN-membrane association of GAP-43(C3S) in 2-BP conditions was not attributable to TGN membrane redistribution since the TGN marker GalNAc-T was not affected under these experimental conditions (Fig. 2E) nor due to an increase in the TGN to plasma membrane vesicular transport of ^N13^GAP-43(C3S), since all experiments were performed at 20°C, a condition which drastically decreases this process [5].

**Figure 3:**
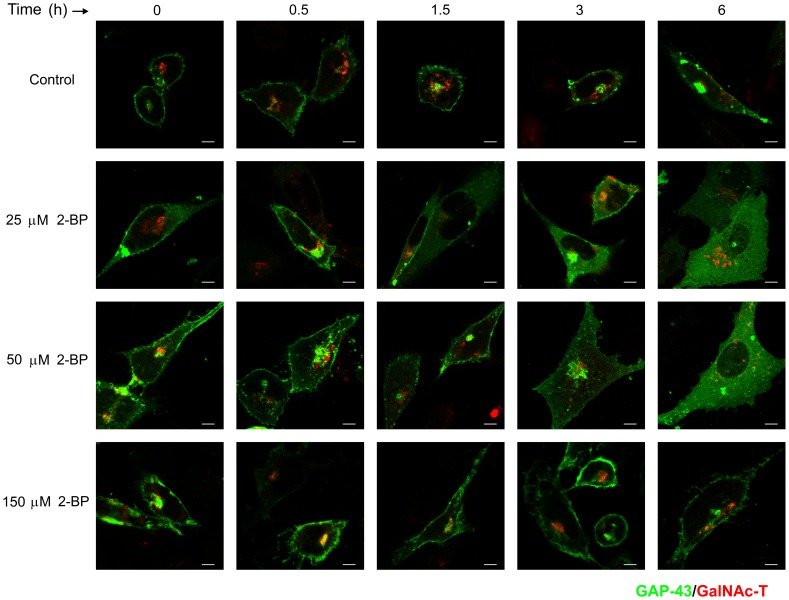
Deacylation kinetic and membrane association of GAP-43 at different doses of 2-BP. CHO-K1 cells coexpressing diacylated GAP-43-YFP and GalNAc-T-CFP were treated with 25, 50 or 150 µM 2-BP or vehicle (DMSO, Control), and the GAP-43 subcellular distribution was analyzed by live cell confocal microscopy at the indicated times. CHX and protein degradation inhibitors were added and maintained in the culture media until the end of each experiment. Representative images show the effect of different doses of 2-BP on the membrane association of GAP-43. The fluorescent signals from YFP and CFP were pseudocoloured green and red, respectively. Scale bars: 5 µm.

Finally, the effect of 2-BP on membrane association of wild-type diacylated GAP-43 was evaluated (Fig. 3). Interestingly, it was observed that in cells treated up to 6 h with 150 µM 2-BP the membrane association of GAP-43 did not significantly change compared to control conditions. In contrast, when cells were treated with 25 and 50 µM 2-BP there was a significant amount of soluble GAP-43 at 3 h, with this being more evident at 6 h. Taken together, these results indicate that 2-BP, in addition to its demonstrated inhibition of protein acylation, can also perturb the protein deacylation in vivo, probably by affecting the catalytic proprieties of thioesterases.

### Enzymological Characterization and Effect of 2-BP on Human APT1 and APT2 Activities

**Figure 4:**
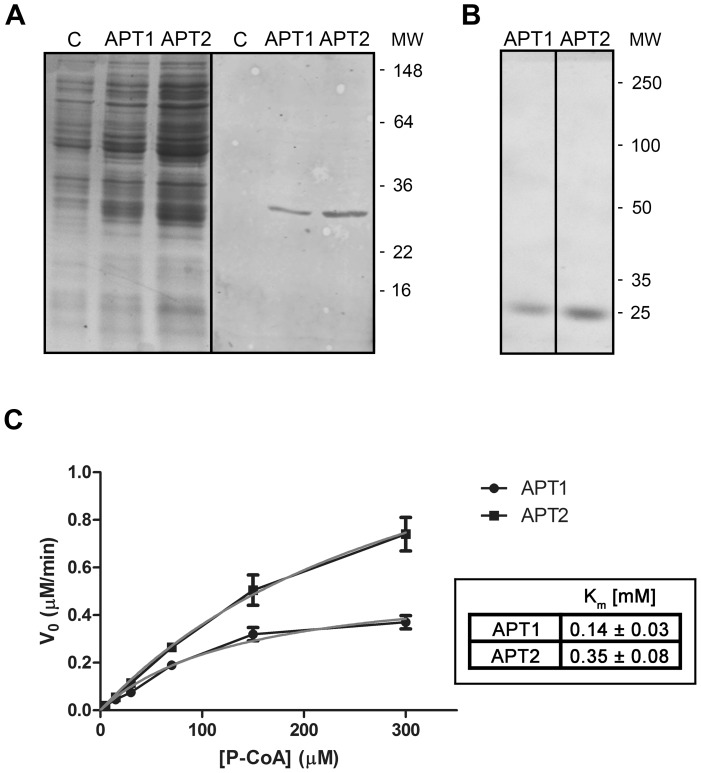
Purification and biochemical characterization of recombinant human APT1 and APT2. **A**) Coomasie blue staining (left) or western blot (right) of supernatant fractions obtained from *E. coli* transformed with His-APT1, His-APT2 or the empty vector (C). Recombinant proteins were analyzed using an antibody to Hisx6 tag. **B**) Coomassie blue staining of recombinant APT1 and APT2 was obtained by affinity chromatography and analyzed by SDS-PAGE. Molecular masses of the markers in kDa are indicated on the right. **C**) The initial rate of palmitoyl-CoA hydrolase activity was measured with 0.5 µg of recombinant APT1 or APT2. The data shown are representative experiments performed in triplicate. Curves were fitted to the Michaelis-Menten rate equation for each data set, and the kinetic parameters are shown in the table.

**Figure 5:**
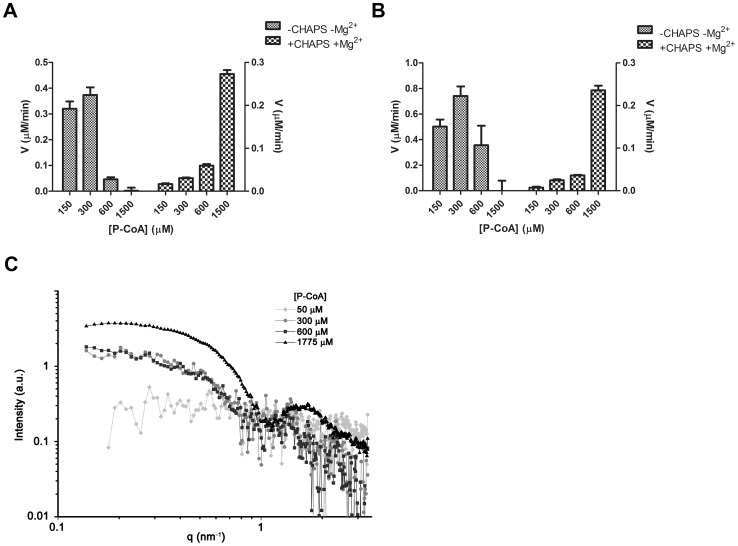
Dose-response curve for APT activities following different concentrations of palmitoyl-CoA and the structural characterization of the substrate by SAXS analysis. The initial rate of palmitoyl-CoA hydrolase activity was measured with 0.5 µg of recombinant APT1 (A) or APT2 (B), both in control conditions (-CHAPS -Mg^2+^, left bars) or in the presence of 7.5 mM CHAPS and 2 mM MgCl_2_ (+CHAPS +Mg^2+^, right bars) at 150, 300, 600 and 1500 µM. Data show the initial rate of the reaction (V, µM/min) at different concentrations of palmitoyl-CoA (P-CoA), which are from representative experiments performed in triplicate. C) SAXS analysis. Palmitoyl-CoA was resuspended in buffer (50 mM Hepes, pH 8.0) at 50, 300, 600 and 1775 µM, and measurement were carried out as indicated in Materials and methods. The figure shows the SAXS raw data (after subtraction of the buffer background and the concentration normalization) for increasing concentrations of palmitoyl-CoA. As can be seen, no noticeable diffraction peak (due to any strong correlation) is observed in any of the curves. The curve for 50 µM palmitoyl-CoA does not display any obvious tendencies. The curves for 300 and 600 µM show increasing intensity at a very low angle, adopting similar slopes and absolute values. The curve at 1775 shows a different behavior with an increment at a low angle, which reached a plateau below 0.3 nm^−1^ with a prominent bump centered at 1.6–1.7 nm^−1^ (very common in bilayers and micelles). In agreement with the wedge-shaped molecular structure, this molecule did not display the global form factor of bilayers, but rather one of the micelles. This is evident from the non-quadratic decay of the intensity as a function of q. The saturation value at low q (Guinieŕs approximation) for the 1775 µM may indicate globular micelles. The clear differences present between the curves at 300–600 µM and the one at 1775 µM is probably due to the fact that the micelles have a different geometry, with the decay at low q values (q<0.5 nm^−1^) having a finite slope closer to an inverse (first power) behavior, suggesting rod-like structures.

**Figure 6:**
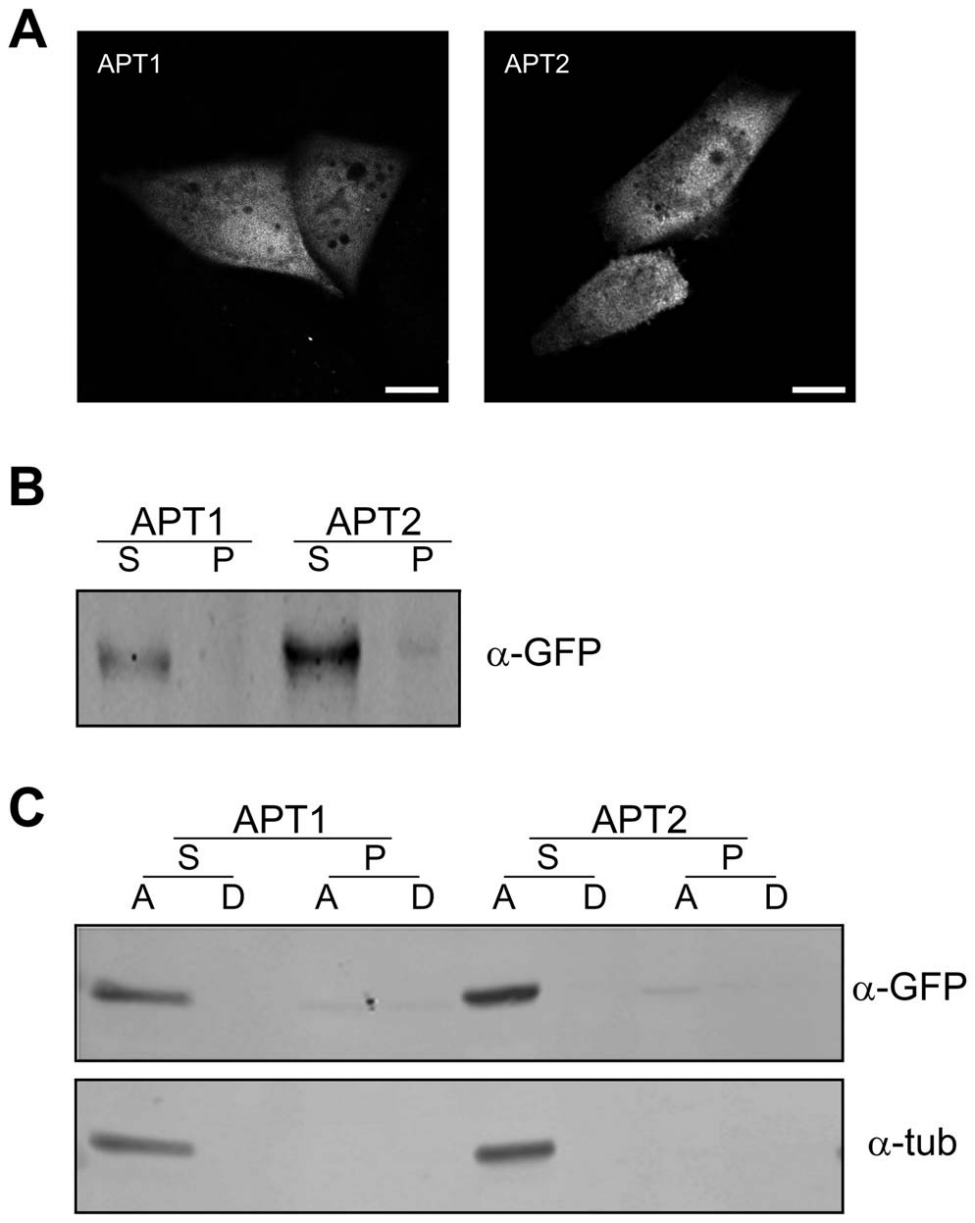
Analysis of acyl-protein thioesterase expression in CHO-K1 cells and biochemical characterization. **A**) CHO-K1 cells transiently expressing APT1-YFP (APT1) or APT2-YFP (APT2) were analyzed by confocal microscopy (pseudocoloured gray). **B**) CHO-K1 cells expressing APT1-YFP (APT1) or APT2-YFP (APT2) were lysed, ultracentrifuged and the supernatant (S) and pellet (P) fractions were recovered. Proteins from these fractions were western blotted with an antibody to GFP (α-GFP). **C**) CHO-K1 cells transiently expressing APT1-YFP (APT1) or APT2-YFP (APT2) were lysed, ultracentrifuged, and the supernatant (S) and pellet (P) fractions were isolated. Buffer containing 1% v/v Triton X-114 was added to the samples and phase separation was induced at 37°C. Proteins from the A (aqueous) and D (detergent) phases were western blotted with an antibody to GFP (α-GFP) and α-tubulin (α-tub). The Triton X-114 partition assay was performed as described by [29]. Note that APT1 and APT2 are mainly present in the aqueous phase of the supernatant (S) fraction, clearly demonstrating their hydrophilic character. Scale bars: 5 µm.

Up to this point, our results indicate that 2-BP perturbs deacylation in living cells. Therefore, we investigated the effect of 2-BP on the activities of recombinant human APT1 and APT2, which are the only two bona fide thioesterases that have been shown to mediate deacylation [19], [29]. First, both human thioesterases were expressed in *Escherichia coli*, purified to apparent homogeneity and biochemically characterized (Fig. 4). As observed in Figure 4A and B, both APT1 and APT2 migrated with an apparent molecular mass of 26 kDa. Then, to test whether both recombinant proteins were enzymatically actives, we evaluated their ability to hydrolyze palmitoyl-CoA, which is a substrate widely used to measure thioesterases. It was observed using a dose-response curves that APT1 and APT2 hydrolyzed palmitoyl-CoA with Km values of 0.14 and 0.35 mM, respectively (Fig. 4C). Interestingly, both enzymes hydrolyzed the monomeric form as well as the micellar state of the substrate palmitoyl-CoA (CMC 100–180 µM), although at higher concentrations of this substrate the deacylation mediated by APTs was inhibited (Fig. 5A and B). These results are of biological relevance, since both thioesterases are mainly expressed in the cytoplasm with a high hydrophilic character (Fig. 6) and might catalyze the hydrolysis of both soluble and membrane-bound substrates. Moreover, it also implies that the structure of the substrate, which depends on the membrane microenvironment, is important in determining the deacylation kinetics, which was also further investigated by Small-angle X-ray scattering (SAXS) analysis (Fig. 5C). As expected, no aggregation of palmitoyl-CoA was observed at 50 µM. However, micellar aggregation was evident at both 300 and 600 µM. Interestingly, at 1775 µM palmitoyl-CoA the SAXS curve changed its shape, indicating that a new kind of aggregate appeared that was compatible with globular micelles (for additional information and analysis see the legend of Fig. 5C). As this occurred in the range of substrate concentration at which the APT activity was lost (Fig. 5A and B), it probably indicates that changes in the structure of the lipid substrate severely affected the enzyme-substrate interaction. However, this phenomenon was not observed when the zwitterionic detergent CHAPS was present in the reaction (Fig. 5A and B), which eventually led to the formation of mixed micelles [13].

**Figure 7:**
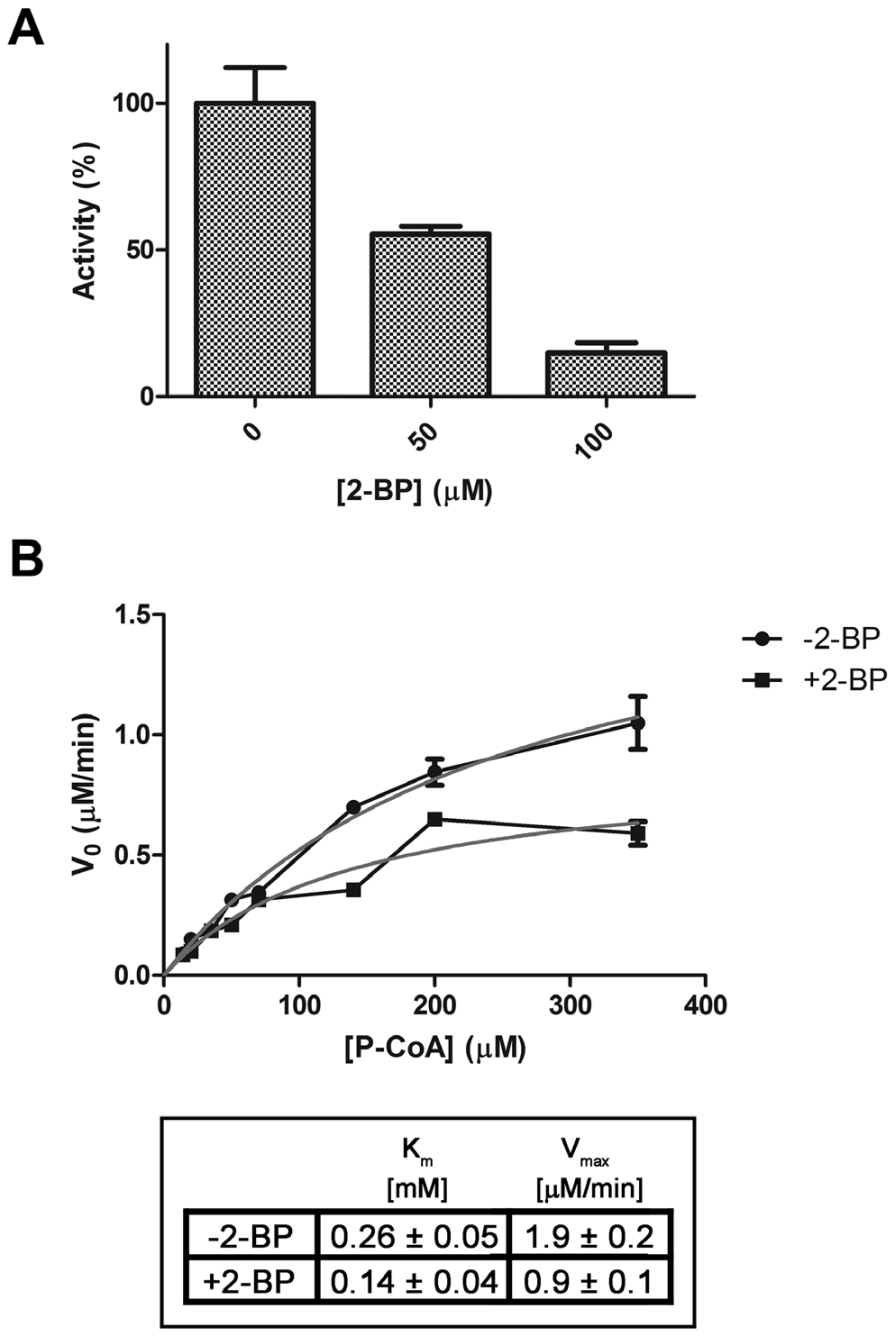
In vitro inhibition of APT1 by 2-BP. **A**) The initial rate of palmitoyl-CoA hydrolase activity was measured with 0.5 µg of recombinant APT1 in the presence of 50 or 100 µM 2-BP or DMSO (vehicle), with the APT1 activity of control condition (vehicle) taken as 100%. **B**) The initial rate of palmitoyl-CoA hydrolase activity (µM/min) was measured with 0.5 µg of recombinant APT1 in the presence of 50 µM 2-BP (+2-BP) or in the presence of DMSO (vehicle, −2-BP). The data shown are representative experiments performed in triplicate. Curves were fitted to the Michaelis-Menten rate equation for each data set. The kinetic parameters (Km and Vmax) are shown in the table below the figure.

After biochemical and enzymological characterization of recombinant human APT1 and APT2, we next evaluated the effect of 2-BP on their enzymatic activities. As shown in Figure 7, a drastic and significant reduction of APT1 activity was observed at 50 and 100 µM 2-BP, with the molecular mechanism of inhibition appearing to be of an uncompetitive type with the apparent Vmax and Km values reduced. Thus, according to this type of enzymatic inhibition, 2-BP would be expected to bind to the enzyme-substrate complex. In the case of APT2, there was a significant effect of 2-BP on its enzymatic activity, reaching 17% and 30% inhibition at 50 and 100 µM 2-BP, respectively.

## Discussion

In the present study we have shown that in vivo treatment with 2-BP, in addition to inhibiting PAT activity, also perturbed the turnover of palmitate moieties on GAP-43 by inhibiting the acyl-protein thioesterases. In particular, it was observed that 2-BP strongly inhibited PAT activity over a range of concentrations from 25 µM to 150 µM. In addition, the TGN-associated fraction of ^N13^GAP-43(C3S), which is highly dependent on the acylation state of the protein, significantly decreased over the time of 2-BP treatment, revealing a half-life of membrane association which is directly proportional to the 2-BP concentration. A similar result was also observed for the diacylated wild-type GAP-43.

The electrophilic α-brominated fatty acid 2-BP, which is highly reactive toward thiols, has been demonstrated to alkylate many membrane-bound proteins through non-specific and non-competitive mechanisms [41]. Although the precise way in which 2-BP exerts this effect is unknown, taking into account the hydrophobic nature of 2-BP, it seems likely that the brominated inhibitor inserts itself into the lipid bilayer and gains access to membrane-bound proteins. In the same way, the soluble proteins that interact with the interface may also be modified by 2-BP. Additionally, 2-BP might act indirectly by modifying the surrounding lipid environment, and hence affect the catalytic properties of integral membrane proteins, such as the PATs. It is also known that 2-BP is converted to 2-BP-CoA inside the cell, which is a non-metabolizable molecule, and the binding of 2-BP to PATs could result in formation of an inhibitor:enzyme complex, thereby affecting the transfer of 2-BP to the acceptor protein. Importantly, 2-BP may also alter lipid metabolism in general, and protein acylation in particular, by reducing the level of intracellular palmitoyl-CoA, which is a necessary donor substrate for palmitoylation [34], [35]. In consequence, evidences indicate that 2-BP exerts multiples, and probably cumulative, effects on the cellular metabolism.

The enzymological analysis performed in this work with recombinant human APTs clearly indicates the significant profile of inhibition of 2-BP. These assays permit us to speculate that the brominated fatty acid is probably perturbing the thioesterase activities through an uncompetitive mechanism resulting from a direct modification of the enzyme, possibly by alkylation.

Recently, it was reported that APT1 and APT2 undergo palmitoylation on cisteine-2 [42], which was suggested to facilitate the steady-state membrane localization and function of these thioesterases. Nevertheless, experimental evidence obtained in our laboratory by biochemical and cell biology assays in CHO-K1 cells has demonstrated that both enzymes are mainly cytosolic with a high hydrophilic character (Fig. 6). Consequently, it is highly probable that non-acylated APTs could transiently associate with the interface in order to exert their catalytic activities. In line with this assumption, recombinant APT1 and APT2 (which are not acylated when expressed in bacteria) hydrolyze the substrate (palmitoyl-CoA) both in its monomeric form or micellar state. In contrast to what has been reported for certain enzymes with membrane-associated substrates (i.e., phospholipases and lipases) [43], [44], we observed that the APTs do not display interfacial activation, as was previously observed for APT1 activity over lyso-phosphocholine [45]. However, various results do indicate that changes in the structure of the lipid substrate drastically affect the APTs-substrate interaction.

Using confocal and video fluorescence microscopy on living cells, we demonstrated that the kinetic of deacylation of the monoacylated ^N13^GAP-43, even at the highest 2-BP concentration, was clearly much faster (minutes) than its wild-type diacylated counterpart (hours). A similar behavior has also been observed in two isoforms of Ras GTPases [11], [15], [46]. The half-life of palmitate on N-Ras (monoacylated) is 20 min and for H-Ras (diacylated) is 2.4 h, with a simple interpretation being that the double acylation is responsible for this longer half-life of palmitate and suggesting that monoacylated species are the preferred substrates for thioesterases. However, it should be mentioned that at steady-state conditions the monopalmitoylated fraction of GAP-43 represents 60% of the total GAP-43 protein [47], suggesting that the double palmitoylation is not an efficient mechanism of acylation in vivo. Consequently, we hypothesize that an important physiological role for APT could be in deacylating single acylated substrates, which should later be in condition to perform another cycle of acylation, or eventually, be sorted for degradation via the ubiquitin-proteasome system [29], [48], [49].

Summing up, our results indicate that 2-BP should be used carefully in the study of the role of PATs in the regulation of protein palmitoylation and function, thus avoiding an erroneous interpretation of kinetic analysis, due to this drug also inhibiting protein deacylation. Nevertheless, controlled experimental conditions using 2-BP could be beneficial in order to “freeze” the turnover of palmitate on palmitoylated proteins, which may permit investigation into the importance of deacylation in the function of these proteins. One such example is Ras, whose deacylation is important for its correct subcellular distribution and function [25], [50]. Our laboratory and other researchers have previously reported that APT1 and APT2 deacylate H-Ras [13], [29], and APTs have been used as molecular targets in the development of drugs to impair Ras signaling [51], [52]. The finding reported in the present work that 2-BP has also the potential to inhibit APT1 and APT2 activity, implies that this moiety can be used as a model for the rational design of new drugs that may be able to modify the oncogenic signaling of Ras, and consequently, might lead to the development of new therapies for cancer.

## Materials and Methods

### Plasmids

The expression vectors pECFP-C1 (where ECFP is enhanced cyan fluorescent protein) and pEYFP-N1 (where EYFP is enhanced yellow fluorescent protein) were from Clontech (CA, USA). Expression plasmids for ^N13^GAP-43(C3S)-YFP, ^N27^GalNAc-T-CFP, APT1-Cherry and APT2-Cherry have been previously described [5], [29], [53].

### Cell Culture and DNA Transfections

CHO-K1 cells (ATCC, Manassas, VA, USA) were maintained at 37°C, 5% CO_2_ in Dulbecco’s modified Eagle’s medium (DMEM) supplemented with 10% fetal bovine serum (FBS) and antibiotics (ATB, 100 µg/ml penicillin and 100 µg/ml streptomycin). Cells grown on Petri dishes were used for both live cell imaging and western blot experiments. These cells were transfected with 0.8–1.5 µg/35 mm dish of the indicated plasmid using cationic liposomes (Lipofectamine, Invitrogen, CA, USA) or polyethyleimine (PEI, Sigma-Aldrich, St. Louis, MO, USA). At 24 h after cell transfeccion, cells were processed for western blot experiments or plated onto Lab-Tek chambered coverglass (Thermo Scientific Nunc, IL, USA), incubated for 24 h, and then used in live cell imaging.

### 2-BP Treatment

A stock solution of 2-BP (0.42 M, Fluka, Sigma-Aldrich, St. Louis, MO, USA) was prepared in DMSO. To analyze the effect of 2-BP on the steady-state subcellular distribution of fluorescent proteins by live cell imaging experiments, CHO-K1 cells were treated with 60 µg/ml CHX (Sigma-Aldrich) and with inhibitors of protein degradation (60 µM cloroquine and 7.5 µM MG132). These inhibitors were added to the culture medium 36 h after plating the transfected cells onto Lab-Tek. 2-BP was added during the time series acquisition.

To analyze the effect of acylation inhibition on the membrane binding properties of ^N13^GAP-43(C3S), 60 µg/ml CHX was incorporated to the culture medium during transfection. Then, 5 h after transfection, 10% FBS was added to the medium and 3 h later 25, 50 or 150 µM 2-BP were also incorporated. After 0.5 h of 2-BP treatment, the CHX was removed. Finally, after 8 h of CHX withdrawal, cells were used in live cell imaging or scraped and used for biochemical experiments.

### Electrophoresis and Western Blotting

Electrophoresis, transfer onto the nitrocellulose membrane and protein immunodetection were performed as described previously [54]. Anti-GFP polyclonal antibody (Roche Diagnostics, IN, USA) was used at a dilution of 1∶800. Antibodies were detected using near infrared fluorescence (Li-COR Biotechnology, Lincoln, NE, USA) with a secondary antibody coupled to IRDye800CW and diluted to 1∶15000 (Li-COR Biotechnology). The relative contribution of the individual bands was calculated using ImageJ software (National Institute for Health, Bethesda, MA, USA).

### Subcellular Fractionation

Cells grown on 60 mm dishes were washed with cold phosphate-buffered saline (PBS, 140 mM NaCl, 8.4 mM Na_2_HPO_4_, 1.6 mM NaH_2_PO_4_, pH 7,5) and harvested by scraping in PBS containing a protease inhibitor mixture (PIM) with 5 µg/ml aproptinin, 0.5 µg/ml leupeptin, and 0.7 µg/ml pepstatin (PBS-PIM). Extracts were centrifuged at 4°C for 5 min at 13000 *g* and resuspended in 400 µl of 5 mM Tris-HCl (pH 7.0) (buffer T) in the presence of protease inhibitors (T-PIM). Pellets were dispersed by repeated pipetting and vortexing. After 30 min of incubation in T-PIM, the pellets were passed 60 times through a 25-gauge needle, and nuclear fractions and unbroken cells were removed by centrifugation at 4°C for 5 min at 600 *g*. The supernatants (S fraction) were then ultracentrifuged at 4°C for 1 h at 400000 *g* using a TLA 100.3 rotor (Beckman Coulter, Inc., CA, USA) before being removed and the pellet (P fraction) resuspended in 400 µl of T-PIM. The proteins in the resulting samples were precipitated with chloroform/methanol (1∶4 v/v) for western blot analyses.

### Expression and Purification of Recombinant APT1 and APT2

The APT1 and APT2 cDNA containing a Hisx6 tag were obtained by RT-PCR using specific primers and RNA from the HeLa and CHO-K1 cells, respectively, and the amplified fragments were cloned into the BamHI/EcoRI sites of the bacterial expression vector pRSET-A (Invitrogen, CA, USA). Transformed *Escherichia coli* cells were grown at 37°C in Luria-Bertani (LB) medium containing 15 µg/ml ampicilin to an optical density of 0.6. Then, a soluble fraction was generated using a high pressure homogenizer (EmulsiFlex, Avestin, Inc., Ottawa, Canada), which was centrifugated at 10000 *g* for 15 min at 4°C. Hisx6 APT1 and APT2 were then purified from the soluble lysate using a Ni^2+^-NTA column (GE Healthcare, Fairfield, VT, USA) according to the manufacturer’s instructions. The purified proteins were then desalted using HiTrap desalting columns (GE Healthcare, Fairfield, VT, USA).

### Confocal Microscopy

Confocal images were collected using an Olympus FluoView FV1000 confocal microscope (Olympus Latin America, Miami, FL) equipped with a multi-line Argon laser (458, 488 and 514 nm) and two helium-neon lasers (543 nm and 633 nm, respectively). YFP was acquired by using laser excitation at 514 nm, a 458/514 nm excitation dichroic mirror, and a 530–560 nm band pass emission filter. Cherry protein was acquired with a laser excitation at 543 nm, a 488/543/633 nm excitation dichroic mirror, and a 560 nm long pass emission filter.

### Live Cell Imaging for Deacylation Kinetic Measurements

For deacylation measurements, cells expressing ^N13^GAP-43(C3S)-YFP were used. The live cell experiments were performed at 20°C on an Olympus FluoView FV1000 confocal microscope to minimize vesicular trafficking of GAP-43. After selection of the cells expressing the protein, images were acquired in the YFP channel for deacylation kinetic measurements using a 63×/1.42 NA PlanApo objective oil immersion (Olympus) with a 3× digital zoom. Image size was 512×512 pixels with a resolution of 165 µm/pixel, with the scan speed being 10 µs/pixel and the pinhole adjusted to obtain an optical slice of 3 µm. Single 3D images were taken at a frequency of 1 min^−1^ for 30 minutes, with the acquisition conditions being optimized in order to acquire the Golgi compartment, to minimize bleaching during acquisition and to sample as quickly as possible the amount of ^N13^GAP-43(C3S) at the TGN [29]. All image analysis and the quantification method were carried out using ImageJ software as described previously [29].

### Palmitoyl-CoA Hydrolase Assay

Palmitoyl-CoA hydrolase assays with purified APT1 or APT2 were performed following a protocol described by Duncan and Gilman [13] with some modifications.

### Small-angle X-ray Scattering (SAXS) Measurements

SAXS was carried out at the SAXS2 beamline at the Brazilian Synchrotron Light Laboratory (LNLS) at Campinas, Brazil. Palmitoyl-CoA was resuspended in buffer (50 mM Hepes, pH 8.0) at 50, 300, 600 and 1775 µM, with the measurement conditions being 5 minutes of exposure, a sample-detector distance of 1 m, 1.5 Å irradiation, 30±1°C. The buffer was measured and subtracted from the signals. Data was collected by a MARCCD and radially integrated by using FIT2D V 12.077 at ESRF. The SAXS curves were generated using the SASFIT free software, and the SAXS intensity was plotted as a function of the scattering vector *q*:
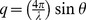
where *λ* is the X-ray wavelength and 2θ is the total scattering angle.

## Supporting Information

Movie S1 **Deacylation of ^N13^GAP-43(C3S) at different doses of 2-BP.** CHO-K1 cells expressing ^N13^GAP-43(C3S)-YFP (pseudocolored green) were incubated in DMEM supplemented with CHX and protein degradation inhibitors at 20°C on the microscope stage, following the experimental procedure described in Fig. 2A. Cells were treated with 25, 50 or 150 µM 2-BP or DMSO (Control), and time series were acquired (1 frame/min) for the following 20 min, as indicated in Materials and methods.
(MP4)
